# Genomic context determines the effect of DNA methylation on gene expression in the gut epithelium of Atlantic salmon (*Salmo salar*)

**DOI:** 10.1080/15592294.2024.2392049

**Published:** 2024-08-16

**Authors:** Aikaterini Katirtzoglou, Søren B. Hansen, Harald Sveier, Michael D. Martin, Jaelle C. Brealey, Morten T. Limborg

**Affiliations:** a Center for Evolutionary Hologenomics, Globe Institute, Faculty of Health and Medical Sciences, University of Copenhagen, Copenhagen, Denmark; b Lerøy Seafood Group ASA, Bergen, Norway; c Department of Natural History, NTNU University Museum, Norwegian University of Science and Technology (NTNU), Trondheim, Norway; d Department of Terrestrial Biodiversity, Norwegian Institute for Nature Research (NINA), Trondheim, Norway

**Keywords:** DNA methylation, salmon, WGBS, RNA-seq, gut, epigenetics, gene expression, aquaculture

## Abstract

The canonical view of DNA methylation, a pivotal epigenetic regulation mechanism in eukaryotes, dictates its role as a suppressor of gene activity, particularly within promoter regions. However, this view is being challenged as it is becoming increasingly evident that the connection between DNA methylation and gene expression varies depending on the genomic location and is therefore more complex than initially thought. We examined DNA methylation levels in the gut epithelium of Atlantic salmon (*Salmo salar*) using whole-genome bisulfite sequencing, which we correlated with gene expression data from RNA sequencing of the same gut tissue sample (RNA-seq). Assuming epigenetic signals might be pronounced between distinctive phenotypes, we compared large and small fish, finding 22 significant associations between 22 differentially methylated regions and 21 genes. We did not detect significant methylation differences between large and small fish. However, we observed a consistent signal of methylation levels around the transcription start sites (TSS), being negatively correlated with the expression levels of those genes. We found both negative and positive associations of methylation levels with gene expression further upstream or downstream of the TSS, revealing a more unpredictable pattern. The 21 genes showing significant methylation-expression correlations were involved in biological processes related to salmon health, such as growth and immune responses. Deciphering how DNA methylation affects the expression of such genes holds great potential for future applications. For instance, our results suggest the importance of genomic context in targeting epigenetic modifications to improve the welfare of aquaculture species like Atlantic salmon.

## Introduction

DNA methylation, the enzymatic addition of a methyl group in a cytosine residue (predominantly in CpG dinucleotides), constitutes a fundamental epigenetic mechanism which has gained significant interest over the past few decades [1]. Together with other epigenetic changes, such as histone modifications and non-coding RNA activity, DNA methylation is involved in regulating gene activity and thus plays a very important role in shaping the phenotype of an organism [2].

DNA methylation is commonly linked to transcription inhibition, especially in the promoter regions of genes [3]. Potential mechanisms leading to gene silencing in hypermethylated genomic areas include reduced binding of transcription factors, promotion of heterochromatin formation, and recruitment of proteins responsible for gene silencing [4]. This relationship appears to depend on genomic areas beyond promoters, with growing evidence supporting that DNA methylation exerts differing influences on gene activities based on the genomic feature [2,5]. Methylation changes downstream of the transcription start site (TSS), in the beginning of the gene, seem to display an antagonistic association with gene expression [6–8]. On the contrary, there is evidence supporting that methylation patterns in the gene body can enhance transcription elongation or have an impact on alternative splicing [9]. All this evidence suggests that DNA methylation’s effect on gene expression is not strictly limited to promoter regions [10]. Finally, the consistent inverse correlation between DNA methylation and gene activity in putative promoter regions has been challenged recently [11], indicating that the association of cytosine methylations with transcriptional regulation is a highly dynamic and context-dependent mechanism [12,13].

Recently, the field of epigenetics has expanded in its exploration to understand how environmental factors influence the growth and health of animals, offering promising applications in animal-based food systems including aquaculture [14]. Evidence suggests that DNA methylation is involved in many biological processes throughout the course of an organism’s life cycle, from embryonic development, immune response to diseases and adaptation to environmental change [15]. Consequently, the potential of epigenome manipulation to enhance successful production in aquaculture is very promising. Specifically, some practical applications that have recently garnered increased attention are the concepts of epigenome editing [16] and epigenetic selection [17].

In the case of Atlantic salmon (*Salmo salar*), a well-established and economically valuable aquaculture species, previous epigenetic studies have focused on changes in the methylome in response to diet composition [18,19], exposure to various environmental stressors [12,20–22], pathogen infection [10,23] or inheritance [24–26]. These studies have offered valuable insights into DNA methylation patterns. Nonetheless, some of them exhibit certain limitations such as the absence of transcriptomic data to elucidate the connection between epigenome modifications and gene expression, which impedes inference of a biologically meaningful context of methylation differences. Other studies that incorporate RNA data only target a limited number of genes that are responsive to external stimuli [12,20]. Finally, the prevalent use of reduced representation bisulfite sequencing methods (e.g., RRBS) introduces a genomic bias towards sequencing primarily GC-rich regions [27].

We study Atlantic salmon with the aim to test the hypothesis that the relationship between DNA methylation and gene expression is indeed dynamic, and dependent upon genomic context. To gain a broad understanding of the functionality of DNA methylation in different genomic locations, we explored methylation and expression at a genome-wide scale rather than focusing on the epigenetic regulation of specific genes. We chose to study farmed Atlantic salmon as it enables new advances to be directly tested in a production relevant system, which provides convenient control of confounding environmental factors such as temperature and diet [28].

Specifically, we tested the connection between DNA methylation and gene expression in Atlantic salmon from a commercial cohort sampling two groups differing in harvest ready size. DNA methylation levels were examined in the gut epithelium using whole-genome bisulfite sequencing (WGBS). We then connected the DNA methylation analysis with gene expression data from RNA sequencing of the same tissue (RNAseq). Combining these data sets allowed us to evaluate the predictive value of differential methylation of specific genomic features on gene expression at a genome-wide scale.

## Materials and methods

### Samples and experimental design

The samples used in this epigenomic analysis were part of a broader hologenomic study in farmed Atlantic salmon [29]. Samples were collected from two sea pens within a commercial production facility close to Bergen, Norway, owned by Lerøy Seafood Group in April 2018, where they received two different commercial diets (Pen1/Feed1 and Pen2/Feed2). The fish were sampled across the entire size distribution of a commercial cohort immediately prior to harvest and were later categorized in size classes based on their gutted weight (range: 0.78–7.83 kg). Small fish were classified as <2.6 kg and large as >4.2 kg. In addition, all samples displayed different levels of infection with an *Eubothrium* cestode. For this study, a total of five large and five small salmon were selected from each pen/feed (*n* = 20). We deliberately selected fish with zero to very low numbers of parasites present in their gut (0–3 visible cestodes) to minimize their potential effect in our analysis (Table S1). For this study, two samples from the distal gut epithelial per individual were taken, one stored in 96% ethanol for WGBS and one stored in RNAlater for RNA-seq. Samples used for this study were obtained as part of the HoloFish project (Norwegian Seafood Research Fund, project no. 901436). This cohort has been described previously [30]. Briefly, gut content samples were originally retrieved from the distal gut, as this is the most immuno-active region of the salmon intestinal tract [31]. Further, evidence shows that pathogens often induce host immune response via epigenetic changes [32] highlighting the applied potential of understanding epigenetic induced immune responses in this gut region.

### Whole-genome bisulfite sequencing

DNA extraction for WGBS was performed using the Qiagen DNeasy® Blood & Tissue Kit 96, following manufacturer’s recommendations and using approximately 20 mg of gut epithelial tissue per sample. DNA concentration was quantified using a NanoDrop 2000. Extracts were then shipped to Novogene for whole-genome bisulfite library preparation and 150-bp paired-end sequencing on the Illumina NovaSeq 6000 instrument.

Raw reads were trimmed for adapters and low-quality base calls using TrimGalore v0.6.6 [33]. After assessing sequencing quality using fastQC v0.11.9 [34], the remaining reads were mapped to the Atlantic salmon reference genome (GCF_905237065.1) using Bismark v0.18.1 [35], which is a wrapper around bowtie2 v2.4.2 [36]. The uniquely aligned reads were deduplicated using Bismark v0.18.1 and after trimming the part of reads subject to methylation bias because of end repair, the methylation information was extracted using Bismark methylation extractor.

### RNA sequencing

The RNA sequencing data was generated and processed as part of a previous study [29]. Briefly, RNA was extracted from 20 mg of gut epithelia per sample using the Quick-RNA™ Miniprep Kit (Zymo Research), following the manufacturer’s recommendations. Extractions were shipped to Novogene for polyA enrichment, mRNA library preparation and 150-bp paired-end sequencing on the Illumina NovaSeq 6000 instrument.

Quality trimming was performed using AdapterRemoval v0.20.4 [37] to remove bases with a Phred score <20 and to trim poly-A tails >8 bp. Sequences shorter than 55 bp and all unpaired reads were excluded from subsequent analyses. Trimmed reads were aligned to the Atlantic salmon reference genome (GCA_905237065.2) using the STAR aligner v2.7.2b [38] with default parameters. As RNA degradation was present in most samples, aligned read counts were adjusted for degradation heterogeneity using DegNorm [39]. Likely due to the RNA degradation, many samples had low mapping statistics to the reference genome, thus all samples with <50% of reads uniquely aligned to the reference were excluded. To reduce noise, only transcripts with at least 10 counts in at least 50% of all samples were included in downstream analyses (filtering was based on all 343 samples included in [29]). After these filtering steps, 17 of the 20 samples used for this WGBS study had sufficient transcriptome data (Table S1). For the differential expression (DE) analysis, genes with fewer than 10 reads across the 17 samples were excluded.

### Analysis of general methylation patterns

Methylation levels from the Bismark analysis were imported to R v4.2.2 and analyzed with R packages dmrseq v1.18.1 [40] and bsseq v1.34.0 [41]. To investigate the general methylation patterns in large and small fish, a principal component analysis (PCA) was performed. Using the R package methylSig v1.10.0 [42], we split the genome in 10kb windows and selected the ones with minimum 100 CpGs, with at least 1X and no more than 30X coverage as input. These parameters were used to ensure a maximum number of CpG sites in each window and to mitigate CpGs in low-complexity regions with high coverage. To explore whether there is a significant difference in methylation profiles between the two size groups, a PERMANOVA test was conducted with the R package vegan v2.6–4 [43]. Finally, we plotted the mean methylation levels around the transcription start site (TSS) of 61,113 annotated genes of the Atlantic salmon genome (assembly Ssal_v3.1, GCF_905237065.1_Ssal_v3.1_genomic.gff) in 50-bp windows to investigate how the CpG methylation level changes in the area surrounding the beginning of genes.

### Differential methylation analysis

We performed a differential methylation analysis between small and large salmon using dmrseq [40]. This program identifies differentially methylated regions (DMRs), a collection of adjacent CpGs that exhibit consistent differences between conditions, in comparison to within-condition variability using a permutation approach [40]. We used DMRs in this study because their methylation state has been shown to have a greater biological inference in terms of a stronger correlation with gene expression, and they can be detected with greater statistical power at lower sequencing depth compared to differentially methylated cytosines (DMCs) [44]. We filtered the Bismark methylation data for the main analysis with different criteria than those used for the PCA. Specifically, we excluded CpG sites with zero coverage in at least one sample before running the program. We tested for DMRs between large and small individuals, while accounting for the effect of feed type in our samples. Large fish were considered as the reference group for the differential analysis metrics. Dmrseq was run with default settings using a lower cut-off value (cutoff = 0.05). DMRs with a *p*-value <0.01 were considered candidate DMRs for downstream analysis. Finally, to visualize the results of the differential methylation analysis, we constructed a volcano plot displaying the *p*-value of each DMR against the raw mean methylation difference (Delta beta) calculated using the dmrseq package.

For the DMR analysis, we chose not to adjust the *p*-value for multiple testing for several reasons. First, considering that this study aims to explore the relationship between DNA methylation and transcription activity rather than finding distinctively different methylation patterns between large and small fish, we opted to analyze several regions considered as candidates to optimize power for testing more general genome-wide methylation patterns. Second, by employing more stringent filtering criteria in the selection process, such as the minimum of 5 CpG sites per region and discarding loci with no coverage in one or more samples, we argue that individual false positives are not likely to affect the general pattern of DMRs. In addition, upon plotting the methylation levels per DMR, we noticed distinctive patterns in many DMRs with a p-value <0.01 between large and small fish which were not deemed statistically significant by the program. This observation suggests that *p*-value adjustment might in this case be overly conservative and overshadow the biological signal in our results. To conclude, while we did not correct for multiple testing, we interpret downstream results with care and only in the light of general patterns as opposed to individual genes due to the potential inclusion of some false-positive DMRs.

### DMR annotation

We annotated the candidate DMRs by dividing the annotation file (assembly Ssal_v3.1, GCF_905237065.1_Ssal_v3.1_genomic.gff) into genomic features relevant for the interpretation of the relationship between DNA methylation and gene expression using BEDTools v 2.31.0 [45], inspired by Saito et al. 2021. The following genomic features were constructed: Distal promoters (P6) defined as 6000 bp upstream of the transcription start site (TSS), Proximal promoters (P1) defined as 1000 bp upstream from the TSS of the gene, Gene start characterized as 1500 bp downstream of the TSS and Gene body as the remaining part of the gene until the Transcription Terminal Site (TTS). Finally, the Transcription Terminal Region (TTR) was defined as 1000 bp downstream of the TTS of the gene (Figure 2c). The candidate DMRs were then mapped on the specified genomic features with BEDTools v2.31.0 *– intersect* [45]. We proceeded to determine the distribution of the candidate DMRs across specified genomic features and intergenic regions. However, as our genomic features vary in length, we calculated a measure of DMR density, by dividing the number of DMRs per feature with its size (bp). An average size of the Gene body regions, of all genes larger than 1500 bp, was used for calculating the DMR density for this feature.

### Differential expression analysis

We investigated differentially expressed genes (DEGs) between large and small fish using the DESeq2 R package [46]. The DE analysis was performed on the Degnorm counts data for 19,500 genes, across the 17 samples. We used large fish as the reference group and accounted for the effect of feed type. DESeq2 automatically adjusts for differences in sequencing depths between samples. To visualize the difference in gene expression between conditions, we constructed an MA plot which displays the log2 fold changes (M) between groups, against the average expression signal (A), per gene. Finally, we created a volcano plot showing the *p*-value of the DE analysis against the log2 fold change for each gene.

### Linking DNA methylation to RNA expression

To explore the association between gene activity and DNA methylation differences, we integrated the paired gene expression and methylation data sets for each relationship between DMRs and genomic features. Gene expression data for all 19,500 genes were utilized to maximize the number of overlaps with the annotated DMRs. Subsequently, we acquired gene IDs corresponding to the detected DMRs to align the proportion of genes that correspond with the RNA-seq data. From the 17 samples with both epigenetic and transcriptomic data, we created a paired dataset for every DMR-gene association. For every sample, we then extracted the mean methylation value for each DMR and the normalized read counts of each gene, as calculated by DESeq2, and plotted each interaction. Pearson’s correlation was calculated for each relationship, considering significance at *p*-values <0.05. A catalog of significant correlations between DMRs and genes was created. Subsequently, gene names or gene symbols from this list were extracted to retrieve broad functional information using various databases, mainly the NCBI gene database (accessed 9 October 2023) and UniProt. In addition, we reviewed relevant literature for every gene separately, to gain a more detailed insight on their respective biological roles.

## Results

A mean (± standard deviation) of 92.3 (±11.3) million epigenetic read pairs were sequenced per sample, which after trimming of adapters and end-repaired bases had a combined mean length per pair of 284.7 (±0.3) bp. The mean alignment rate was 66.2% (±2.2%) and hereafter 19.3% (±1.7%) duplicated reads were removed resulting in methylation information from 49.2 (±5.6) million reads corresponding to a coverage of 5.0X (±0.6). The mean methylation level in CpG context was 78.4% (±0.8%) and 0.4% (±0.04%) outside CpG context (Table S1).

### Genome-wide methylation analysis in gut epithelium of Atlantic salmon

**Figure 1.**
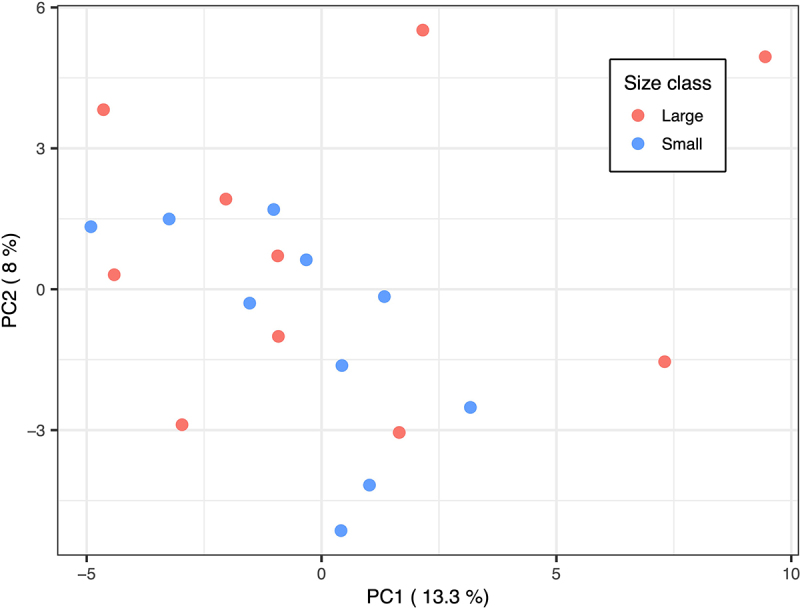
Principal component analysis (PCA) of methylation levels for 90,616 genomic tiles (12,076,477 CpG sites). Individuals are plotted on the two first principal components and colored by size. (PERMANOVA test: R-squared = 0.048, p-value = 0.46).

The PCA based on the methylation level of 90,616 genomic tiles (12,076,477 CpG sites) with a mean coverage of 3.67X did not indicate genome-wide significant differences in methylation patterns between large and small fish in the gut tissue (Figure 1). The absence of significantly different methylation profiles between the two size classes was also supported by a PERMANOVA test, which showed that only 4.8% of the variation in DNA methylation levels is explained by the size class factor (R-squared = 0.048, *p*-value = 0.46).

The differential methylation analysis was conducted on 20,419,191 CpG sites. We detected 1,057 candidate DMRs (*p*-value <0.01) from a total of 111,201 tested regions for downstream analysis. The candidate DMRs had a mean length of 774 bp and contain 22 CpG sites on average. The methylation levels of the candidate DMRs were higher in large fish. Specifically, 576 of the selected candidate DMRs were hypermethylated (54.5%) and 481 (45.5%) were hypomethylated in large fish as shown in the volcano plot (Figure S1a).

### Annotation of DMRs on specific genomic features

**Figure 2.**
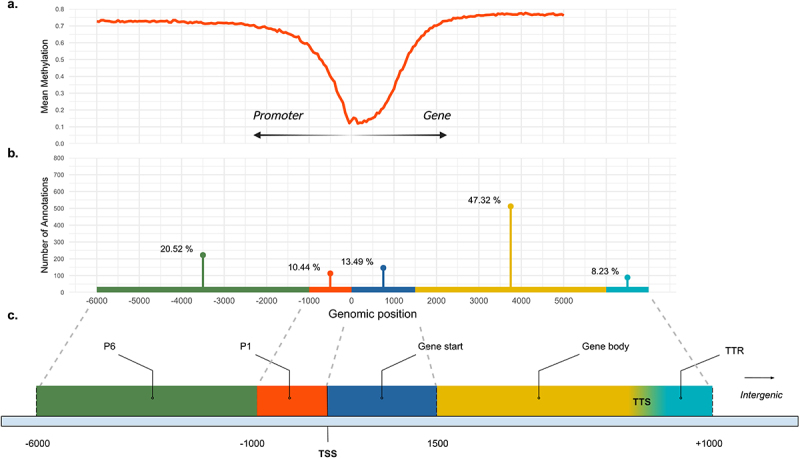
(a) Mean methylation level of CpGs in 50-bp windows located from 6000 bp upstream to 5000 bp downstream of the annotated genes’ transcription start sites. The decline in methylation levels observed upstream and downstream of the TSS was considered in the definition of the genomic features. (b) DMR distribution on specific genomic features after their annotation on the reference genome (c) definition of genomic features. Distal promoter (P6) and proximal promoter were defined as 6000 bp and 1000 bp upstream from the transcription start site (TSS) respectively. Gene start was defined as the first 1500 bp of each annotated gene and the remaining part was referred to as gene Body. Transcription terminal region (TTR) was defined as 1000 bp downstream the TTS. The height of the bars shows the number of annotations (N_A_) linked to each genomic feature.

We observed a gradual decrease in the mean methylation levels around the transcription start site (TSS) of genes. This decline in methylation values not only in the region upstream of the TSS, but also within the gene, was considered when dividing the reference genome into relevant genomic features (Figure 2a).

Out of 1,057 candidate DMRs 779 DMRs were overlapping genomic features of 938 unique genes, while the remaining 278 DMRs were located in intergenic regions. Annotating DMRs on genomic features can be challenging. A single DMR may cover multiple features, or different genes’ genomic features may overlap leading to multiple DMRs associated with one feature. To preserve information on each unique DMR–gene interaction, we counted each interaction as one annotation (*N*_*A*_ = number of annotations). However, we also assigned each DMR to one feature (*N*_*DMRs*_ = number of DMRs) based on a hierarchy: P1 > Gene start > P6 > Gene body > TTR > Intergenic regions. This allowed us to obtain a more precise view of the DMR distribution on different genomic features (Table 1).

**Table 1.** The number of annotations and DMRs linked to different genomic features; one DMR can map on multiple features of the same gene, while different DMRs can be associated with a single gene. We calculated the number of annotations (N_A_) to capture every single dmr–gene relationship. The number of DMRs mapped on genes smaller than 1500 bp is shown in parentheses in the N_A_ column. The hierarchy: proximal promoter > gene start > distal promoter > gene body > transcription terminal region (TTR) > Intergenic regions was used to report the distribution of the total number of DMRs and assign it to a specific genomic feature. The DMR density per genomic feature (N_DMRs_/Size of gene feature in bp) is shown in the last column.

Genomic feature	N_A_	N_DMRs_	N_DMRs_/Size of gene feature (bp)
Distal promoter (P6)	222	140	0.028
Proximal promoter (P1)	113	109	0.109
Gene start	137 (+9)	74	0.049
Gene body	512	423	0.014
TTR	89	33	0.033
Intergenic	-	278	-
Total	1082	1057	-

The majority of the annotations (*N*_*A*_) were assigned to the Gene body (47.32%) and the Distal promoter (20.52%), followed by Gene start (13.49%) and Proximal promoter (10.44%), with fewer in the TTR (8.23%). The number of annotations assigned to the Gene start feature includes the nine DMRs located within genes smaller than 1500 bp (Figure 2b, Table 1). Regarding DMR density in different genomic features, we observed that the rate of DMRs/bp is higher in the Proximal promoters compared to the other features, followed by Gene start, which suggests that potentially our candidate DMRs are enriched in the genomic area around the TSS of genes (Table 1).

### Connecting DNA methylation to gene expression

The DE analysis did not yield any significantly differentially expressed genes (FDR <0.05) between small and large salmon, although 91 genes had a raw *p*-value <0.01, with 56 of them upregulated in large individuals and 35 downregulated in large individuals (Figure S1b and Figure S2). However, gene expression data were integrated in this study to understand the direct influence of DNA methylation on transcriptional regulation among genomic features. Hence, all 19,500 genes were utilized in downstream analysis.

Linking methylation and expression data yielded 22 significant DMR–gene relationships. First, we discarded a portion of the annotated DMRs, due to lack of overlap with the available RNA-seq data. Specifically, out of 19,500 genes with available RNA-seq data, 418 overlapped with genes found from DMR annotation, corresponding to a total of 396 unique DMRs with paired RNA-seq data. We correlated mean methylation levels with normalized counts for each of the 478 gene–DMR interactions. A total of 22 DMRs showed a significant correlation (*p*-value <0.05) with the expression of 21 paired genes (Table 2), of which some of the 22 DMRs overlapped with more than one gene feature. To assign one DMR to one genomic feature, we applied the following hierarchy: Proximal promoter > Gene start > Distal promoter > Gene body > Transcription Terminal Region (Table S2).

**Table 2.** Number of annotations and DMRs linked to different genomic features after overlap with rna-seq data; N_A_ was utilized to report every single association of dmr-gene feature as previously described. The number of DMRs mapped on genes smaller than 1500 bp is shown in parentheses in the N_A_ column. The hierarchy: proximal promoter (P1) > gene start > distal promoter (P6) > gene body > transcription terminal region (TTR), was used to report the distribution of the total number of DMRs (N_DMRs_). The number of significant correlations between normalized counts of a gene and mean methylation of its corresponding DMR was calculated per genomic feature. The proportion of significant correlations by total correlations was also calculated for every feature. The last column shows the number of negative significant correlations.

Genomic feature	N_A_	N_DMRs_	Significantly correlated with gene expression	Proportion of significant correlations (%)	Number of negative correlations
Distal promoter (P6)	93	71	2	2.8	0
Proximal promoter (P1)	38	38	7	18.4	6
Gene start	44 (+1)	27	4	14.8	1
Gene body	249	225	9	4	8
TTR	53	35	0	0	0
Total	478	396 (418 genes)	22 (21 genes)		15

The significant correlations between gene expression and methylation levels were more frequently associated with the Proximal promoter genomic feature followed by the Gene start, with 18.4% and 14.8% of the DMRs located on the respective features significantly connected to gene expression. We found a relatively low proportion of significant correlations linked to Gene body and Distal promoter features, 4% and 2.8%, respectively. Finally, we did not find any significant correlations of DMRs associated with the TTR. Our results indicate that while more DMRs may be located in Distal promoters and Gene body features, the influence on transcriptomic regulation is more pronounced when methylation occurs in the Proximal promoter and Gene start (Table 2).

### Exploring the interplay between DNA methylation and gene expression in a genomic feature-specific context

**Figure 3.**
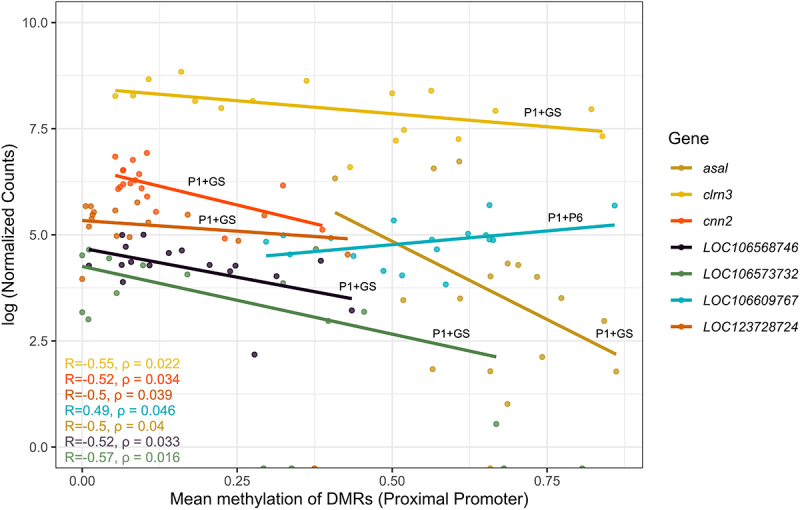
Correlation plot of gene expression against mean methylation of the seven DMRs with significant correlations between gene expression and methylation levels located in proximal promoter (P1). Six out of seven DMRs on P1 extended downstream in the gene start feature (P1+GS) and one DMR extended upstream towards the distal promoter (P1+P6). All P1+GS DMRs displayed an inverse relationship with gene expression of their corresponding genes, whereas P1+P6 was positively correlated with gene expression. Pearson’s correlation was calculated between the raw normalized counts and mean methylation levels, for each dmr–gene interaction. Normalized counts were log-transformed on the y axis for visual clarity due to differences in the mean of normalized counts between genes.

Upon examining the significant DMR-gene correlations, we found that the methylation and gene expression levels were predominantly negatively correlated, especially for DMRs located in proximity to the TSS. Of the 22 significant methylation – gene expression correlations, 15 exhibited negative relationships (Table 2). This result aligns with the notion that increased methylation levels are associated with suppressed gene expression. Moreover, we observed that methylation seems to exert a different output on gene activity depending on the genomic feature. Specifically, both DMRs located in Distal promoters showed positive correlations with gene expression (Fig S3a). However, eight of nine DMRs located on Gene body features were negatively associated with gene expression (Fig S3c). Three out of four DMRs found on Gene start features had positive relationships with gene expression (Fig S3b). Finally, among the DMRs located on the Proximal promoter, the methylation levels of six out of seven exhibited an inverse correlation with gene activity. We observed that these six DMRs also extend to the Gene start feature and are hence overlapping with the TSS (Figure 3). Notably, the only DMR on the Proximal promoter displaying a positive correlation value was primarily situated within the Distal promoter, thus in greater distance upstream of the TSS. Hence, our results suggest that the repressive effect of DNA methylation may be strongly associated with the area around the beginning of genes in both directions. In our dataset, this area constitutes the Proximal promoter and Gene start features. Contrary to this, the effect of DNA methylation on the gene expression is more unpredictable in other genomic features.

### Investigation of biological functions of genes significantly linked to DMRs

We explored the functions of the 21 genes significantly correlated with 22 DMRs and found that they are involved in diverse biological mechanisms. Exploring the phenotypic implications of DNA methylation is a challenging process, especially when genes under investigation are similarly expressed. Among the 21 genes significantly correlated with 22 DMRs, only seven displayed well-established gene symbols. A total of 11 genes had only weak associations with biological functions (‘LOC’ prefix) and three genes were ‘Uncharacterized’ according to the NCBI database (Table 3). The 21 genes were primarily involved in immune response pathways against pathogens (*gzma, lsm4, gimap4, asal, rab5a)* [47–51]. Other functions included muscle contraction (*ppp1r14d, cnn2)* [52,53], cholesterol transfer activity (*gramd1a*) [54], energy generation in muscle cells (*camk2g*) [55] and inflammation response (*nlpr3*) [56]. We also identified the gene *atp10d* (Phospholipid-transporting ATPase VD), which has been shown to improve insulin sensitivity and reduce the chance of developing obesity in mice fed a high-fat diet [57]. Additionally, we found DMRs located within the Gene body of genes linked to muscle development and lipid accumulation. Notably, these genes included Acetyl CoA carboxylase (*acaca*), one of the main lipogenic genes in fish [58], which plays a key role in lipid deposition in muscle tissue, and the Ubiquitin-conjugating enzyme E2 G1 (*ube2g1*), a hub gene shown to play an important role in muscle development and degradation in Nelore cattle [59]. In addition, we found that the majority of the 22 DMRs connected with the 21 genes were hypermethylated in large fish (Table 3). Overall, despite discovering two genes (*acaca, ube2g1*) with differential methylation signatures that may be linked to size-related functions, their expression levels in the gut are not significantly different between large and small fish. Thus, their phenotypic consequences remain speculative.

**Table 3.** Genes with significant relationship to DMRs per feature; in parentheses are aliases for genes that begin with the prefix ‘LOC.’ The gene *gimap4* is included two times in the list, as two different DMRs are associated with it. Genes with an asterisk (*) were uncharacterized. The arrows indicate the methylation status of the corresponding DMRs, with ↑ displaying hypermethylation in large individuals and ↓ display hypomethylation in large individuals.

Genomic feature	Gene
Distal promoter (P6)	LOC106593568 (*gimap4*) ↑, LOC106569220 (*sc61g*) ↑
Proximal promoter (P1)	*clrn3*↓, *asal*↓, LOC106609767 (*krr1*) ↑, LOC106568746 (*nlpr3*) ↑, *cnn2*↑, LOC106573732 (*nudt21*) ↑, *LOC123728724 ↑
Gene start	*ppp1r14d*↓, *gzma*↓, LOC106593568 (*gimap4*) ↑, *LOC106581915 ↑
Gene body	*atp10d*↑, LOC106582397 (*gramd1a*)↑, LOC106603271 (*acaca*)↑, LOC106603929 (*ube2g1*)↓, *eml3*↓, LOC106613629 (*lsm4)*↑, LOC106576966 (*camk2g*) ↑, *LOC106584195↑, LOC106588886 (*rab5a*) ↑
TTR	-

## Discussion

The outcome of this study has provided insight into how genomic context determines the influence of DNA methylation on gene expression levels in Atlantic salmon. We examined genome-wide DNA methylation levels in correlation to gene expression data in the gut epithelial tissue of Atlantic salmon and identified 22 significant associations. Our findings suggest a consistent signal of methylated levels around the TSS being negatively correlated with gene expression, however receding either upstream or downstream of the beginning of genes yielded more variable relationships including both negative and positive correlations.

### Interpreting general methylation patterns

Based on the broader hologenomic study, our samples were a part of [29], the source of variation in the transcriptome data was better explained by the size class and feed type than other known factors. For the methylome data, we constructed a PCA (Figure S4) of fish grouped by sex, which showed no differentiation. While sex was reported for the individuals, most had not reached the level of maturity, introducing a level of uncertainty in these observations [30]. Thus, we explored the differential methylation and expression signals between large and small fish while adjusting for feed type. These results suggest that sex does not confound our methylation-based results in gut epithelium.

We selected size as a factor for our analysis because there is growing evidence linking immune responses and epigenetic modifications [10,23,60], and our previous study showed a correlation between size and health status in salmon [61]. Additionally, research in other organisms demonstrates that body size variation is related to DNA methylation patterns [62].

The general methylation patterns between large and small individuals did not significantly differ, which could be attributed to various factors. First, it is widely accepted that epigenetic modifications are tissue-specific signals [63]. Hence, investigating methylation patterns in the specific tissue type that closely corresponds to the factor under study could promote a more accurate assessment of the relationship between the factor and its associated epigenetic changes [64]. The differentiation analyses in our workflow were based on the size variation observed in our dataset, but utilizing tissues like muscle, blood, or liver might better capture and reflect growth variation. Second, body size constitutes a highly complex polygenic trait shaped by multiple factors, including genetic background and environmental stimuli, thus epigenetic signatures may be masked by other functions. Finally, the transcriptome activity is not exclusively controlled by DNA methylation but can be influenced by a combination of other epigenetic mechanisms like histone modification or non-coding RNAs, it is rather challenging to assess which of these mechanisms primarily impacts gene expression levels and thus explain phenotypic traits.

However, upon plotting the methylation levels for each DMR, we noticed distinctive patterns between large and small fish for many DMRs with a p-value <0.01. Therefore, we opted to analyze these regions with caution, considering them as putative DMRs. Taking the above into account, the goal of this study was mainly to investigate the genomic context of the epigenome–transcriptome cross talk, rather than directly exploring the effect of DNA methylation on size variation in Atlantic salmon.

In terms of genome-wide methylation levels, we observed a distinct decrease around the TSS. This pattern has been described previously, as transcription start sites usually coincide with CpG Islands, broadly termed as a collection of hypomethylated CpG sites, clustered together around the beginning of genes [9]. The annotation step revealed that 73.6% of our candidate DMRs were enriched in genes (Gene start, Gene body) and associated genomic features (Distal and Proximal promoters, TTRs). This result reveals that DMRs are characterized by a non-random genome-wide distribution. Further, CpG sites often aggregate together in specific genomic locations and their methylation levels correlate, forming DMRs [65] which can be detected even at low coverage [66].

The mean coverage of 3.67X in our analysis, for CpGs used in the PCA, is at the low end. However, the sequencing effort in this analysis is much higher than similar studies in salmon [67,68]. We cannot directly compare our WGBS data with RRBS approaches used in the aforementioned studies, however we believe that the use of whole genome data along with interpreting methylation signals using DMRs instead of DMCs highlights the value of our analysis in salmon. In addition, we do not necessarily believe that discarding sites with low coverage would lead to more robust analysis but would rather limit it to fewer random sites, reducing our ability to infer genome wide patterns. Importantly, the trade-off between number of biological replicates and sequencing depth was benchmarked using a downsampling approach in Ziller et al. [66]. Here, the authors demonstrated that coverage as low as 2X could be sufficient to find robust DMRs, supporting that our results confidently reflect the general DNA methylation patterns in our dataset. In addition, DMRs which also encompass larger genomic regions are often considered as more attractive methylation markers due to exhibiting more biological significance compared to individual CpG sites [69]. There is also evidence suggesting that changes in methylation levels of DMRs can have a more substantial impact on the regulation of nearby genes [44].

### Genomic context determines influence of methylation on gene expression

Our results suggest that the output of DNA methylation–gene expression interplay varies, depending on the genomic feature in salmon. Out of the 22 significant DMR–gene relationships from our analysis, we found at least one associated with every defined gene feature apart from the TTR (Table 2). This observation suggests that DNA methylation’s influence on transcription is more perplexing than initially perceived, as there is concrete evidence that its influence extends beyond promoter regions [5,70,71]. However, Proximal promoters and Gene start features exhibited the highest proportion of significant relationships, as shown in Table 2, despite their smaller size compared to Distal promoters or Gene bodies. This finding underpins their exceptional role in regulating gene activity. This conclusion was further supported by the high DMR density in P1 and Gene start compared to other genomic features. Conversely, the flank region in the 3’ end of a gene (TTR in our study) has been reported to be influential on expression, since it might overlap with downstream enhancers [10], which deviates from our finding.

Most of the significant correlations with gene expression were negative (15 negative correlations out of 22). A general negative association trend, independent of genomic features between DNA methylation and gene expression, was also reported in the head kidney and liver in Atlantic salmon, in a previous study [10]. However, considering these correlations within separate genomic features allowed us to observe the epigenome–transcriptome interactions in a higher resolution. Methylation around the TSS, specifically DMRs extending from Proximal promoters to Gene start features, was consistently inversely correlated with gene expression. This agrees with the classical dogma of DNA methylation acting as a suppressor of gene activity, especially when located in putative promoter regions [3]. There is growing evidence that DNA methylation at the beginning of the gene is negatively correlated to transcription, specifically for the first exon [6], or the first intron [5], shifting the interest from only exploring promoter methylation. Together, this evidence suggests that high methylation levels close to the TSS, either upstream or downstream, are possibly inducing gene silencing.

Subdividing the Gene start region into smaller genomic features comparable with other studies, could theoretically provide deeper insights into the role of genomic context in DNA methylation regulation. However, we opted for a lower-resolution definition for two reasons. First, we believe that by this approach we do not risk ignoring a broader, equally important pattern that still needs to be established. Second, our dataset lacks significant DMRs (four) to further split the Gene start meaningfully. Hence, using broader definitions allows us to better utilize our current data, while a larger dataset would be necessary for more detailed exploration.

We observed both negative and positive methylation-gene expression relationships in regions further upstream or downstream for DMRs that do not coincide with TSS. Specifically, we found positive correlations in promoters (all DMRs on Distal and one in Proximal promoters). More evidence is surfacing supporting that hypermethylation of CpGs in promoter regions can actually enhance gene activity, thus questioning the canonical narrative of the inverse relationship between the two mechanisms [11]. In the Gene start and Gene body features, positive and negative correlations prevailed, respectively; however, we found a mix of them for each genomic feature. High levels of gene body methylation in humans have been associated with increased gene expression activity in the active X chromosome [70], and there is evidence that gene body methylation can indeed maintain transcription activity by preventing histone modifications [72]. A previous study in mammals has demonstrated that both positive and negative correlations exist between tissue-specific DMRs (t-DMRs) and transcription, probably mediated by two distinct mechanisms which employ a different set of transcription factors (TFs) [73]. Gene body methylation in cancer cells has also shown conflicting signals on expression even within the same genes, for neighboring CpG sites [74].

In conclusion, our results suggest that the genomic context seems to play a crucial role in methylation-mediated gene expression. Even though the conclusions of this analysis are relevant to the gut tissue in salmon, the notion that the relation of DNA methylation and gene expression depends on the gene feature, independently of tissue and species, has already been demonstrated by [5]. In addition, our findings indicate that DMR methylation in proximity to the TSS exerts a more pronounced effect on transcription and exhibits consistent negative relationships. However, both positive and negative epigenetic signatures on gene activity are found upstream towards Distal promoters and downstream within the gene bodies, highlighting the need to validate methylation patterns with paired gene expression data.

### Exploring genes linked to methylation changes

We focused on genes significantly linked to our candidate DMRs to study the relevance of genome-wide methylation changes at the transcriptional level of specific genes. However, by not identifying DEGs in our dataset, we are not able to decipher the functional implications of these 22 DMRs and thereby we interpret this section prudently.

A significant proportion of genes seems to be involved in the immune response system, which was partially expected, as the gut constitutes a barrier against pathogens and often mediates immune responses like inflammation [75]. The rest of the 21 genes, whose expression levels are regulated by their DNA methylation status, displayed a broad set of functions related to lipid metabolism, inflammation response and muscle activity (section 3.5) suggesting that DNA methylation is crucial for the observed phenotypic variation. The gut microbiome has been shown to be implicated in many physiological processes in fish such as development of immunity, nutritional metabolism and disease resistance [76]. We chose to study the gut because of increasing evidence of a so-called epigenome-microbiome axis [23,77–79] which constitutes the dynamic interaction between the host epigenome and the microbiome. This evidence suggests that epigenetic modifications might be pronounced in the gut. Hence, by studying the intestinal tissue, we argue that the 21 genes showing significant correlations with DMRs in this study serve as interesting candidates for understanding how the salmon host may actively control its intestinal microbiome. However, testing this hypothesis by identifying direct associations between genes regulated by DNA methylation and intestinal microbiota of salmon, is beyond the scope of this study.

Genes *ube2g1* and *acaca* were differentially methylated between large and small individuals, with functions potentially connected to size variation. Interestingly, *ube2g1*, which initiates muscle degradation, was found to be down-regulated in nelore cattle individuals with high rib-eye muscle area [59]. In our study, however, this gene was hypomethylated and with increased expression levels in large fish. The *acaca* gene, which plays a crucial role in synthesis of long-chain fatty acids, was hypermethylated and with decreased expression level in large fish. The *acaca* gene has already been shown to act as a potential upstream epigenetic regulator of lipid biosynthesis in salmon responding to dietary changes, by methylation modifications in its promoter region [19]. Finally, it is worth mentioning that the two DMRs associated with *acaca* and *ube2g1* are located in their respective Gene body features, which, as discussed above, seems to exert unpredictable effects on gene expression compared to DMRs that coincide with the TSS.

Taking into account the aforementioned complexities, it is evident that interpreting DNA methylation patterns in a biologically meaningful context is not straightforward. However, it is likely that DNA methylation impacts the expression of genes that influence size including the two genes *acaca* and *ube2g1* detected in this study.

### Final remarks

The emerging field of epigenetics holds promise for novel techniques in manipulating gene expression with profound implications for research or therapeutic purposes [16,80]. However, a crucial step toward the successful development of epigenome editing tools involves deepening our understanding of how different epigenetic modifications, such as histone posttranslational modifications or ncRNAs, shape biological functions. In this study, we investigated the DNA methylation landscape in Atlantic salmon, a species of considerable importance for aquaculture. Epigenetics could revolutionize various aspects of production systems by refining selective breeding techniques, enhancing our understanding of adaptation mechanisms and developing strategies for disease resistance. One potential application in aquaculture is the use of nutritional stimuli to trigger epigenetic modifications and tailor economically important traits [81]. Emerging developments in CRISPR/Cas systems will likely be of great value in aquaculture practices, as they offer the potential to alter the epigenetic marks, like DNA methylation, of targeted genomic features [32]. We believe that for the successful implementation of such precision-editing tools, understanding how DNA methylation affects gene expression in different genomic positions is imperative.

## Supplementary Material

TableS1_Supplementary_revised.xlsx

Supplementary_Material_revised.docx

## Data Availability

Raw WGBS and transcriptomic sequencing reads are available at the European Nucleotide archive (ENA) under project PRJEB64334. Sample metadata and ENA accessions are provided in Supplementary Table S1. Scripts used for the analysis are publically available at GitHub under the repository name ‘EpiG-salmon-2024’ (https://github.com/katkat-ri/EpiG-salmon-2024).
