# Supplementary material for: Genomic context determines the effect of DNA methylation on gene expression in the gut epithelium of Atlantic salmon (Salmo salar)
Source: Epigenetics. 2024 Aug 16;19(1):2392049. doi: 10.1080/15592294.2024.2392049 (PMC11332636; doi:10.1080/15592294.2024.2392049)
Supplement: Supplementary_Material_revised.docx [file KEPI_A_2392049_SM6325.docx]

**Supplementary Material**

Genomic context determines the effect of DNA methylation on gene expression in the gut epithelium of Atlantic salmon (*Salmo salar*)

Aikaterini Katirtzoglou, Søren B. Hansen, Harald Sveier, Michael D. Martin, Jaelle C. Brealey, Morten T. Limborg

**Table S1**: Fish metadata and summary statistics of the sequencing results of the 20 samples; Fish ID, feed type, pen gutted weight, sex, size class (based on gutted weight). The rest of the columns display: cestode presence, availability of WGBS and RNA-seq data and mapping rate of the RNA-seq data on the reference genome. Number of raw WGBS reads, percentage of reads aligned on the reference genome, percentage of duplicated alignments that were removed, number of aligned reads, mean length of read pairs (forward plus reverse reads after trimming of adapters and bases suffering from m-bias) and coverage assuming genome length of 2.8GB. Percentage of cytosine methylation in the three types of methylation context: CpG, CHG and CHH (where H corresponds to A, T or C). Mean and standard deviation (SD) were calculated for each WGBS metric from all samples. In the last columns, ENA accession numbers for the WGBS and RNA-seq data.

**Table S2**: Annotation of DMRs that significantly correlate with gene expression data. A DMR index from 1:1057 is shown along with annotation details: chromosome, start and end position. Each DMR is assigned to one gene feature based on the hierarchy: Proximal promoter (P1) > Gene start > Distal promoter (P6) > Gene body > Transcription Terminal Region (TTR). If the DMR mapped on more than one feature, they are displayed in parentheses. The gene name or gene symbol of the corresponding gene are reported and finally the gene description is provided.

| ***Index*** | ***Chr*** | ***Start*** | ***End*** | ***Region*** | ***Gene*** | ***Gene description*** |  |
| --- | --- | --- | --- | --- | --- | --- | --- |
| 5 | NC_059442.1 | 24845749 | 24846552 | Gene Start | *ppp1r14d* | Protein Phosphatase 1 Regulatory Inhibitor Subunit 14D |  |
| 11 | NC_059442.1 | 32043124 | 32043830 | Gene Body | *atp10d* | Phospholipid-transporting ATPase VD |  |
| 49 | NC_059442.1 | 114253534 | 114253650 | P1 (Gene start) | *clrn3* | Clarin-3 |  |
|  |  |  |  |  |  |  |  |
| 59 | NC_059442.1 | 131386265 | 131386540 | Gene Start | *gzma* | Granzyme A |  |
| 83 | NC_059443.1 | 19124744 | 19125662 | P1 (Gene start) | *asal* | Mannose-specific lectin |  |
|  |  |  |  |  |  |  |  |
| 99 | NC_059443.1 | 37910815 | 37911054 | Gene Body | LOC106582397 (*gramd1a)* | protein Aster-A |  |
| 217 | NC_059445.1 | 46488125 | 46488446 | Gene Body | LOC106603271  (*acaca*) | Acetyl CoA carboxylase |  |
| 238 | NC_059445.1 | 81066653 | 81067130 | Gene Body | LOC106603929  (*ube2g1*) | ubiquitin-conjugating enzyme E2 G1 |  |
| 309 | NC_059448.1 | 13279939 | 13280827 | Gene Body | *eml3* | Echinoderm microtubule-associated protein-like 3 |  |
| 327 | NC_059448.1 | 55500238 | 55501794 | P1 (P6) | LOC106609767 | KRR1 small subunit processome component homolog |  |
|  |  |  |  |  |  |  |  |
| 383 | NC_059451.1 | 16704450 | 16704988 | Gene Body | LOC106613629 | U6 snRNA-associated Sm-like protein LSm4 |  |
| 470 | NC_059452.1 | 101762879 | 101763475 | P6 | LOC106593568  (*gimap4*) | GTPase IMAP family member 4-like |  |
| 471 | NC_059452.1 | 101767822 | 101769295 | Gene Start (Gene Body) |  |  |  |
| 587 | NC_059455.1 | 10096444 | 10097881 | P1 (Gene start) | LOC106568746  (*nlpr3*) | NACHT, LRR and PYD domains-containing protein 3-like |  |
| 590 | NC_059455.1 | 12911018 | 12912950 | P1 (Gene Start, P6) | *cnn2* | Calponin 2 |  |
| 605 | NC_059455.1 | 25603865 | 25604398 | P6 | LOC106569220  (*sc61g*) | protein transport protein Sec61 subunit gamma |  |
| 688 | NC_059457.1 | 31478783 | 31479733 | P1 (Gene Start) | LOC106573732  (CPSF5, nudt21) | cleavage and polyadenylation specificity factor subunit 5 |  |
| 741 | NC_059459.1 | 16620095 | 16622029 | Gene Body | LOC106576966  (*camk2g*) | calcium/calmodulin-dependent protein kinase type II subunit gamma |  |
| 749 | NC_059459.1 | 53133245 | 53133819 | P1 (Gene start) | LOC123728724 | Uncharacterized |  |
| 877 | NC_059462.1 | 19626247 | 19627152 | Gene Start | LOC106581915 |  |  |
| 937 | NC_059464.1 | 14215905 | 14216247 | Gene Body | LOC106584195 |  |  |
| 1018 | NC_059468.1 | 29456476 | 29457101 | Gene Body | LOC106588886  (*rab5a*) | ras-related protein Rab-5A |  |


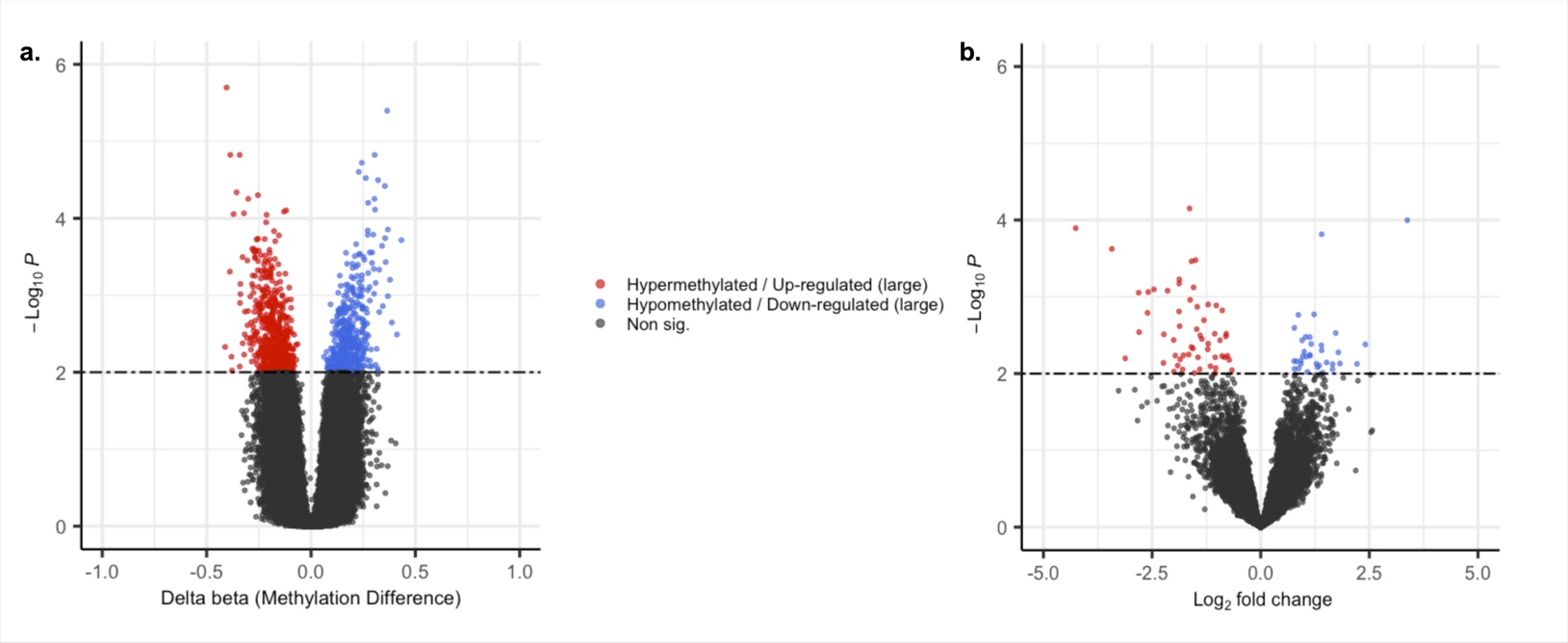


**Fig. S1.** Volcano plots a) Results of differential methylation analysis for 111,201 DMRs. The y axis shows the logarithmic raw *p*-value and the x axis the mean methylation difference (delta beta) of each DMR between large and small individuals. Out of 1,057 DMRs with a *p*-value < 0.01, 576 were hypermethylated and 481 were hypomethylated in large fish, marked in red and blue respectively. b) Results of differential expression analysis for 19,500 genes. The y axis shows the logarithmic raw *p*-value and the x axis the log2 fold change in expression of each gene between large and small individuals. Out of 91 genes with a *p*-value < 0.01, 56 were up-regulated and 35 down-regulated in large fish, marked in red and blue respectively. However, all 19,500 genes were utilized in downstream analysis with the aim of maximizing the number of overlaps with the methylation data.

**Fig. S2.** MA plot for the DE analysis of 19,500 genes. An MA plot displays the magnitude of the differential gene expression on the y axis, measured as a log2 fold change against the average expression of the gene across all samples measured as the mean of normalized counts. Out of 91 genes with a *p*-value < 0.01, 56 were up-regulated and 35 down-regulated in large fish, marked in red and blue respectively. The DE analysis did not yield any significant, differentially expressed genes between large and small salmon, based on the FDR correction.

**Fig. S3.** Correlation plots of gene expression against mean methylation of DMRs located on specified genomic features. a) Both DMRs located in Distal promoter regions displayed a positive correlation with gene expression levels b) Three out of four DMRs located on the Gene Start region displayed a positive correlation with gene expression. c) Eight out of nine DMRs located in Gene body regions displayed a negative correlation with gene expression. Two different DMRs were associated with the LOC106593568 gene; one was located on its Distal promoter region and one on the Gene start. Pearson’s correlation was calculated between the raw normalized counts and mean methylation levels, for each DMR-gene interaction. Normalized counts were log-transformed in the y axis for visual clarity due to differences of the mean of normalized counts between genes.

**Fig. S4.** Principal component analysis (PCA) of methylation levels for 90,616 genomic tiles (12,076,477 CpG sites). Individuals are plotted on the two first principal components and colored by sex.
